# Supplementary material for: The steroid-hormone ecdysone coordinates parallel pupariation neuromotor and morphogenetic subprograms via epidermis-to-neuron Dilp8-Lgr3 signal induction
Source: Nat Commun. 2021 Jun 7;12:3328. doi: 10.1038/s41467-021-23218-5 (PMC8184853; doi:10.1038/s41467-021-23218-5)
Supplement: Supplementary file 1 — Supplementary Information [file 41467_2021_23218_MOESM1_ESM.pdf]

Supplementary Information for

The steroid-hormone ecdysone coordinates parallel pupariation neuromotor and morphogenetic subprograms via epidermis-to-neuron Dilp8-Lgr3 signal induction

Fabiana Heredia*, Yanel Volonté*, Joana Pereirinha*, Magdalena Fernandez-Acosta, Andreia P. Casimiro, Cláudia G. Belém, Filipe Viegas, Kohtaro Tanaka, Juliane Menezes, Maite Arana, Gisele A. Cardoso, André Macedo, Malwina Kotowicz, Facundo H. Prado Spalm, Marcos J. Dibo, Raquel D. Monfardini, Tatiana T. Torres, César S. Mendes, Andres Garelli†, Alisson M. Gontijo†

* These authors contributed equally to this manuscript.
†Correspondence to: agarelli@inibibb-conicet.gob.ar (A.G.) and alisson.gontijo@nms.unl.pt (A.M.G.)

This file contains:

Supplementary Fig. 1 – 12
Supplementary Tables 1-2.

Supplementary Fig. 1.

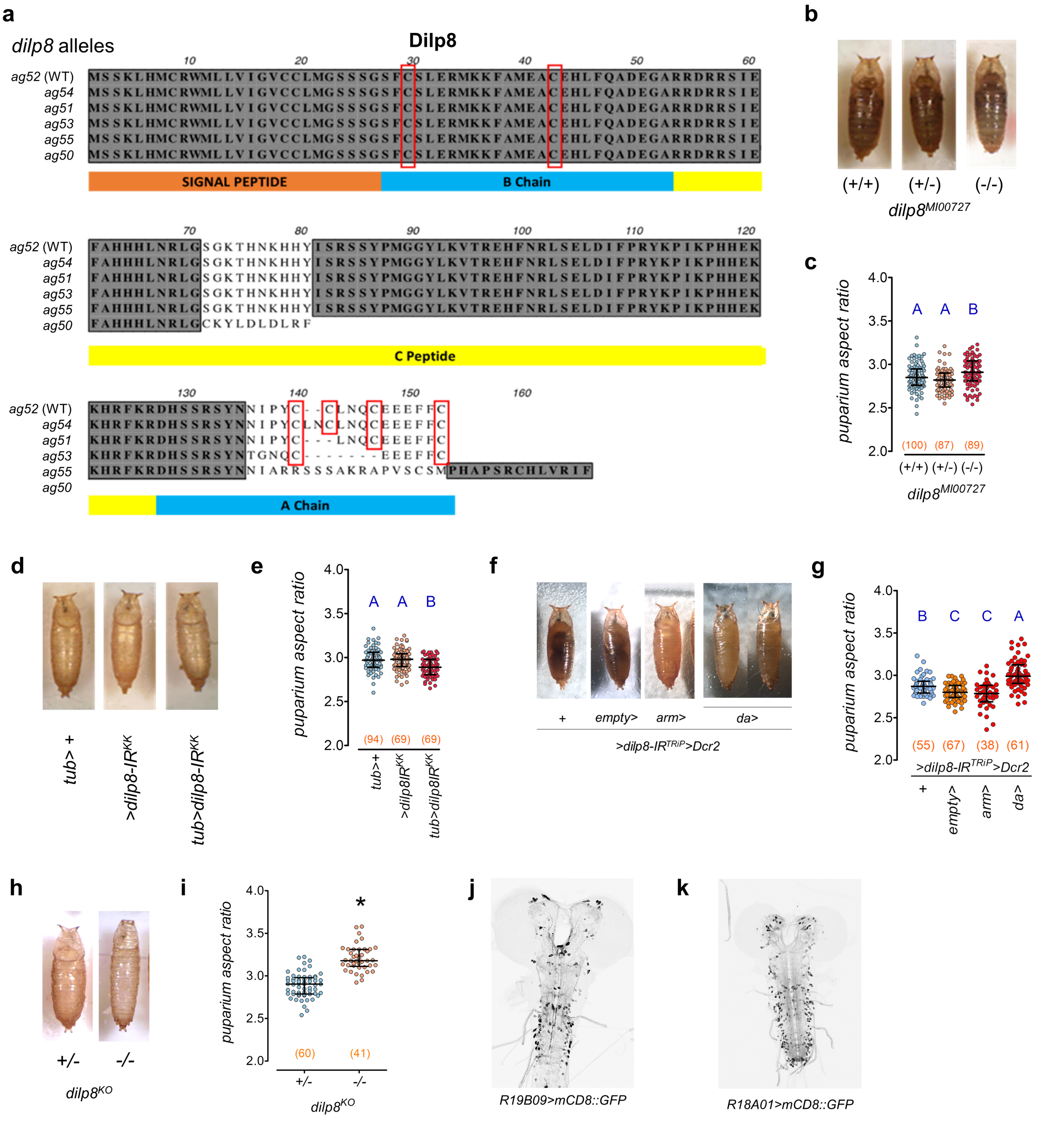

Supplementary Fig. S1: CRISPR-Cas9-generated *dilp8* mutants and phenotypes of hypomorphs and a knockout.

a Protein sequence of one wild type *dilp8*^{ag52} and five *dilp8* mutant alleles (*dilp8*$^{ag50-55}$) generated by CRISPR-Cas9 mediated germline-mutagenesis directed to the 3' end that encodes the A-chain with the highly conserved cysteines essential for Dilp8 activity (highlighted with red boxes). **b** Photos of puparia of animals carrying the hypomorphic allele *dilp8*^{MI00727} in homozygosis (-/-) or heterozygosis (+/-). (+/+), WT controls. **c** Puparium aspect ratio (AR) of animals with the same genotype as **b**. Shown are dot plots of puparium AR. **d** Photo of puparia of animals ubiquitously-expressing RNAi against *dilp8* (*tub>dilp8-IR*^{KK}) and controls (*tub> and UAS-dilp8-IR*^{KK}). **e** Knockdown of *dilp8* in *tub>dilp8-IR*^{KK} animals does not increase AR. Shown are dot plots of puparium AR. **f** Representative photos of puparia from the depicted genotypes. **g** Knockdown of *dilp8* with a second RNAi line, *UAS-dilp8-IR*^{TRiP} in combination with *UAS-Dcr2,* increases AR when driven by *da*>, but not *arm*> ubiquitous

drivers. Shown are dot plots of puparium AR. **h** Representative photos of puparia from the depicted genotypes. **i** *dilp8^{KO}* mutants have increased puparium AR. Shown are dot plots of puparium AR. **j** FlyLight[53] confocal projections of *R19B09>* and **k** *R18A01>* driving expression of *UAS-mCD8::GFP*. Statistics (full details in Supplementary Table 2): **c, e, g, i** Dots: one animal. Horizontal bar, median. Error bars: 25-75% percentiles. **c, g** Dunn's test. **d** Student-Newman-Keuls test. **h** Student's t-test . Same blue letters, *P*>0.05. **g** * *P= 6.31 E-18*. (N) number of animals (orange).

Supplementary Fig. 2.

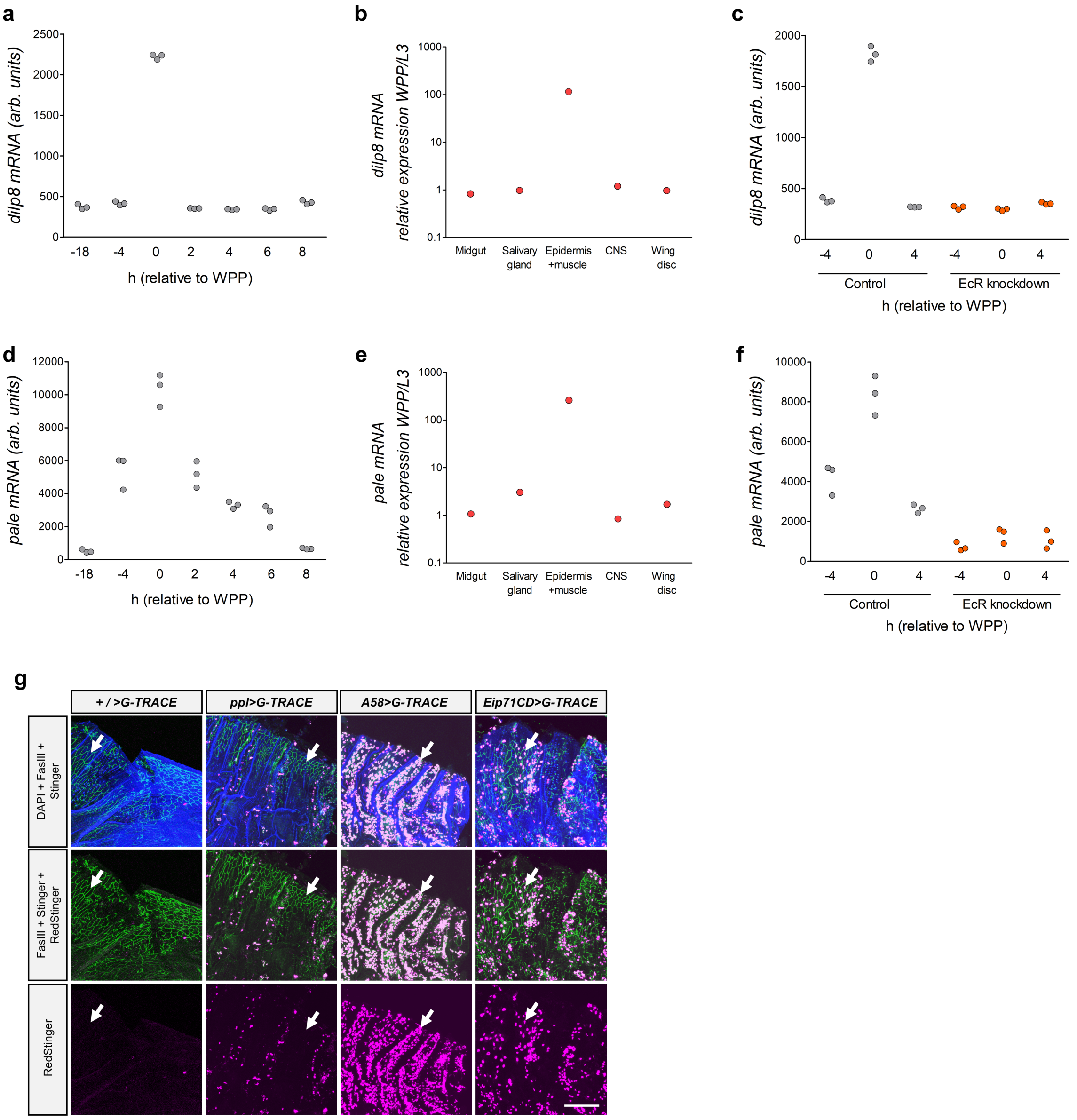

Supplementary Fig. 2: *dilp8* and *pale* expression profiles and G-TRACE labelling of epidermal cells.

a *dilp8* mRNA expression peaks in white prepupa (WPP T0). Shown are results obtained from microarray expression experiments[55]. **b** *dilp8* mRNA is upregulated in the WPP-T0 carcass (muscle and epidermal cells). Shown are results obtained from microarray expression experiments[54]. **c** Ecdysone receptor (EcR) knockdown prevents *dilp8* upregulation at WPP T0. Shown are results obtained from microarray expression experiments[55]. **d** *pale* mRNA expression peaks in WPP T0, but with a different dynamics than *dilp8* (a)[55].e *pale* mRNA is upregulated in the WPP-T0 carcass (muscle and epidermal cells). Shown are results obtained from microarray expression experiments[54]. **c** Ecdysone receptor (EcR) knockdown prevents *pale* upregulation at WPP T0. Shown are results obtained from microarray expression experiments[55]. **g** Confocal Z-projections of immunofluorescence images of epidermal cells of the WPP T0 cuticle stained with anti-Fasciclin (FasIII; cell membranes, green) and the G-

TRACE system to label past (Stinger=EGFP::NLS, nuclear green) or current (RedStinger=DsRed::NLS, nuclear magenta) expression of *ppl*>*, A58*>, and Eip71CD> GAL4 drivers. Blue, DAPI counterstain (nucleus) and cuticle autofluorescence. *A58*> and *Eip71CD*> drive current (nuclear magenta) and past (nuclear green) expression in the large nuclei of cuticle epidermal cells of WPP T0, *A58*> being the strongest GAL4 driver. *ppl*> drives no or only sporadic current or past cuticle epidermal-cell expression at this developmental stage. White arrows, regions of cuticle epidermal cells. Representative images of at least 3 animals per genotype. Scale bar, 200 μm.

Supplementary Fig. 3.

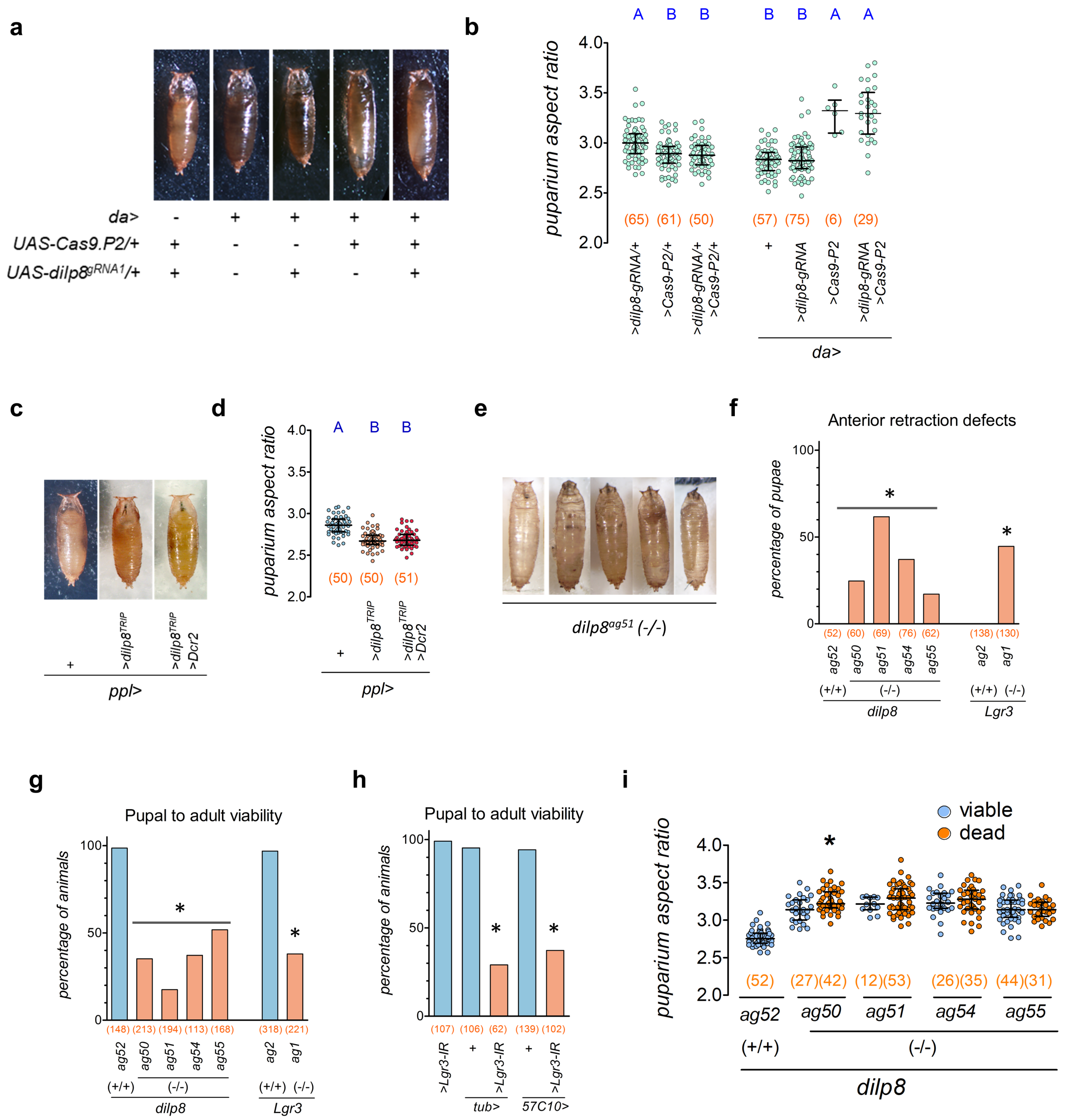

Supplementary Fig. 3: Tissue-specific CRISPR-Cas9 and reduced pupal viability of *dilp8* and *Lgr3* loss of function.

a Cas9 alone affects puparium morphology. Shown are photos of animals ubiquitously-expressing (*da-GAL4, da>*) *UAS-Cas9P2* and/or *UAS-dilp8^{gRNA1}* for targeted somatic mutagenesis of *dilp8.* **b** Dot plots of puparium aspect ratio following ubiquitous *Cas9* and/or *dilp8^{gRNA1}* expression. **c** Representative photos of puparia from the depicted genotypes. **d** Silencing of *dilp8* in the fat body does not increase the puparium aspect ratio (AR). *ppl>/+* animals in **c, d** are from the same batch as used in Fig. 2e, f. **e** Pictures of representative puparia of *dilp8* mutant animals displaying increasingly severe anterior retraction defects. **f** Percentage of puparia with defective anterior retraction. **g** Pupal viability is reduced in animals lacking *dilp8* and *Lgr3* or **h** following ubiquitous (*tub>*) or panneuronal (*R57C10>*) *Lgr3* knockdown. **i** Dot plots of puparium aspect ratio in a series of *dilp8* mutant alleles (*ag50, 51, 54, and* 55) or WT control (*ag52*), according to pupal viability (blue, viable; orange, dead).

Pupal viability was not associated with puparium AR, except in one of the four assayed *dilp8* mutant alleles (*ag50*). Statistics (full details in Supplementary Table 2): **b, d, i** Dots: one animal. Horizontal bar, median. Error bars: 25-75% percentiles. Same blue letter, $P>0.05$. **b,d** Dunn's test. **f-h** Binomial tests with Bonferroni corrections. *$P<0.001$. **i** Bonferroni's test for multiple comparisons, *$P<0.05$ (N) number of animals (orange).

Supplementary Fig. 4.

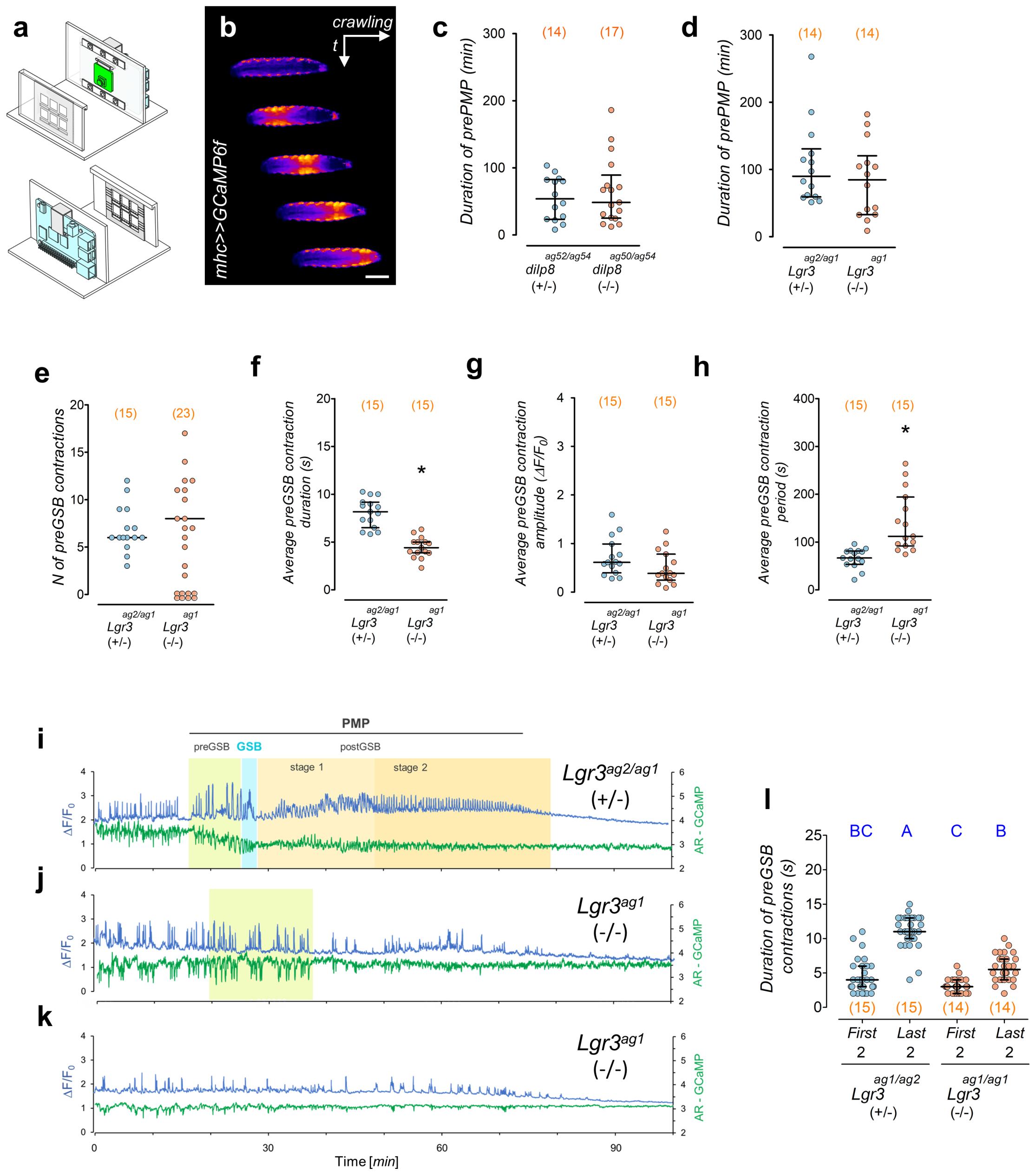

Supplementary Fig. 4: Effects of *dilp8* and *Lgr3* on pre-PMP and pre-GSB.

a Schematics of the pupariation monitoring device. **b** Time-lapse of a larva expressing the muscle-specific calcium sensor, mhc>>GCaMP, showing peristaltic contractions during larval crawling. Representative profile of 6 monitored animals. **c** Dot plots showing pre-pupariation motor program (pre-PMP) duration in *dilp8* and **d** *Lgr3* mutant and control animals. **e** Dot plots showing the number of pre-GSB contractions in *Lgr3* mutant and control animals. **f** Dot plots showing average duration, **g** amplitude, and **h** period of pre-GSB contractions in *Lgr3* mutants and controls. **i** Temporal profile of mhc>>GCaMP fluctuations (blue) and AR (green) in control animals. **j, k** Same as (i), but in *Lgr3* mutant animals. *Lgr3* mutants either show pre-GSB-like contractions (**j**), or not (**k**). **l** Duration of the first and last two pre-GSB contractions in *Lgr3* mutants and controls. *Lgr3* mutants fail to increase the duration of the pre-GSB contractions

with time. Statistics (full details in Supplementary Table 2): **c-h** Dots: one animal. **l** Dots: one contraction. **c-h, l** Horizontal bar, median. Error bars: 25-75% percentiles. **c, d, g, h** Mann-Whitney test. **e, f** Student's t-test. **c-h** *P*=0.86, 0.26, 0.058 (animals without contractions excluded), 1.51E-08, 0.106 and <0.001, respectively. **P*<0.001. **l** Same blue letters P>0.05, Dunn's test. (N) number of animals (orange). Scale bar, 1 mm.

Supplementary Fig. 5.

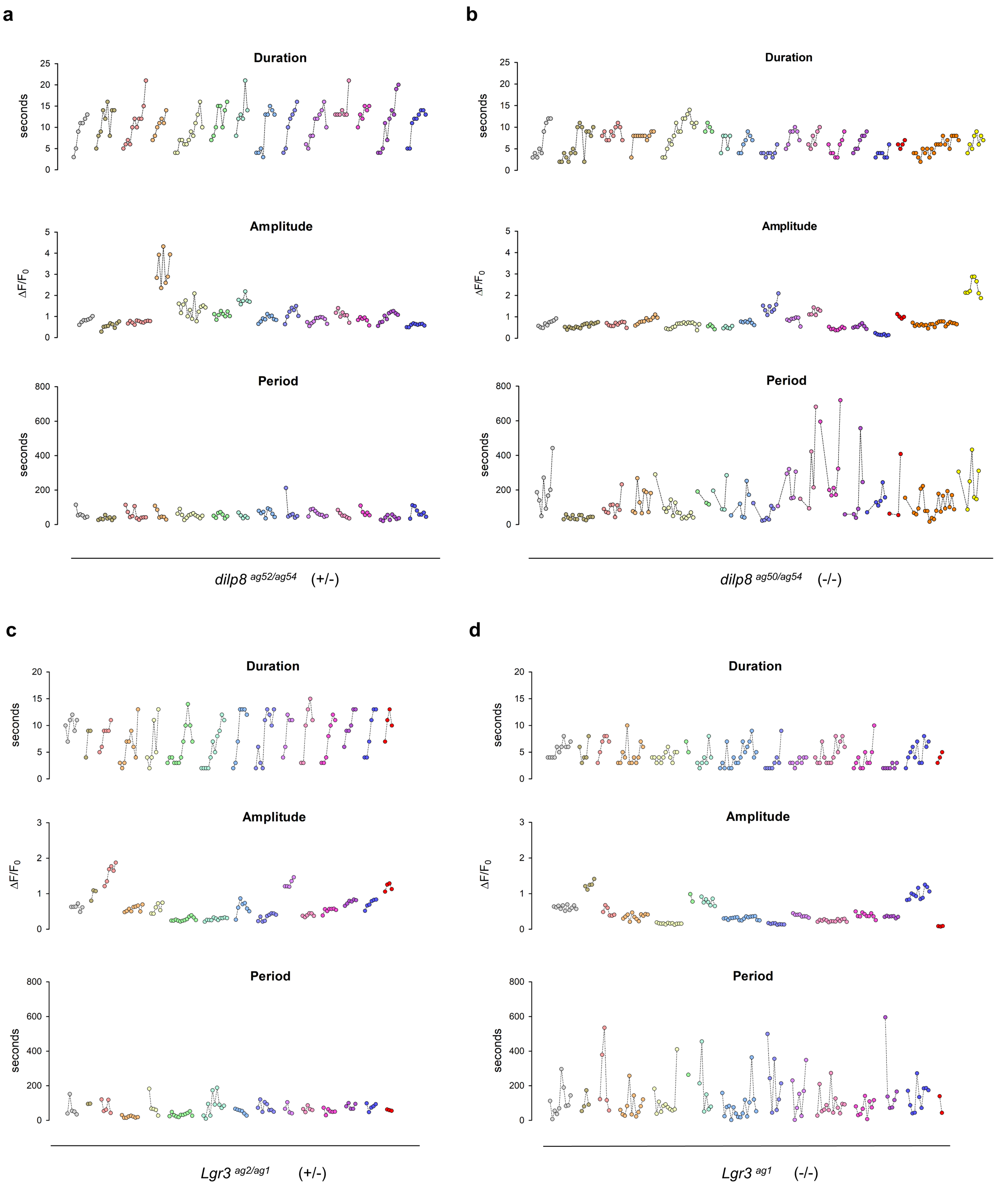

Supplementary Fig. 5: Effects of *dilp8* and *Lgr3* on pre-GSB peak parameters.

a Duration, amplitude, and period of pre-GSB contractions (as determined by mhc>>GCaMP-fluorescence peaks) in WT animals of the *dilp8*$^{ag52/ag54}$ *(+/-)* genetic background, **b** *dilp8* mutant animals [*dilp8*$^{ag50/ag54}$ *(-/-)*], **c** WT animals of the *Lgr3*$^{ag2/ag1}$ *(+/-)* genetic background, and **d** *Lgr3* mutant animals [*Lgr3*$^{ag1/ag1}$ *(-/-)*]. Dots connected with a line represent consecutive contractions of one larva.

Supplementary Fig. 6.

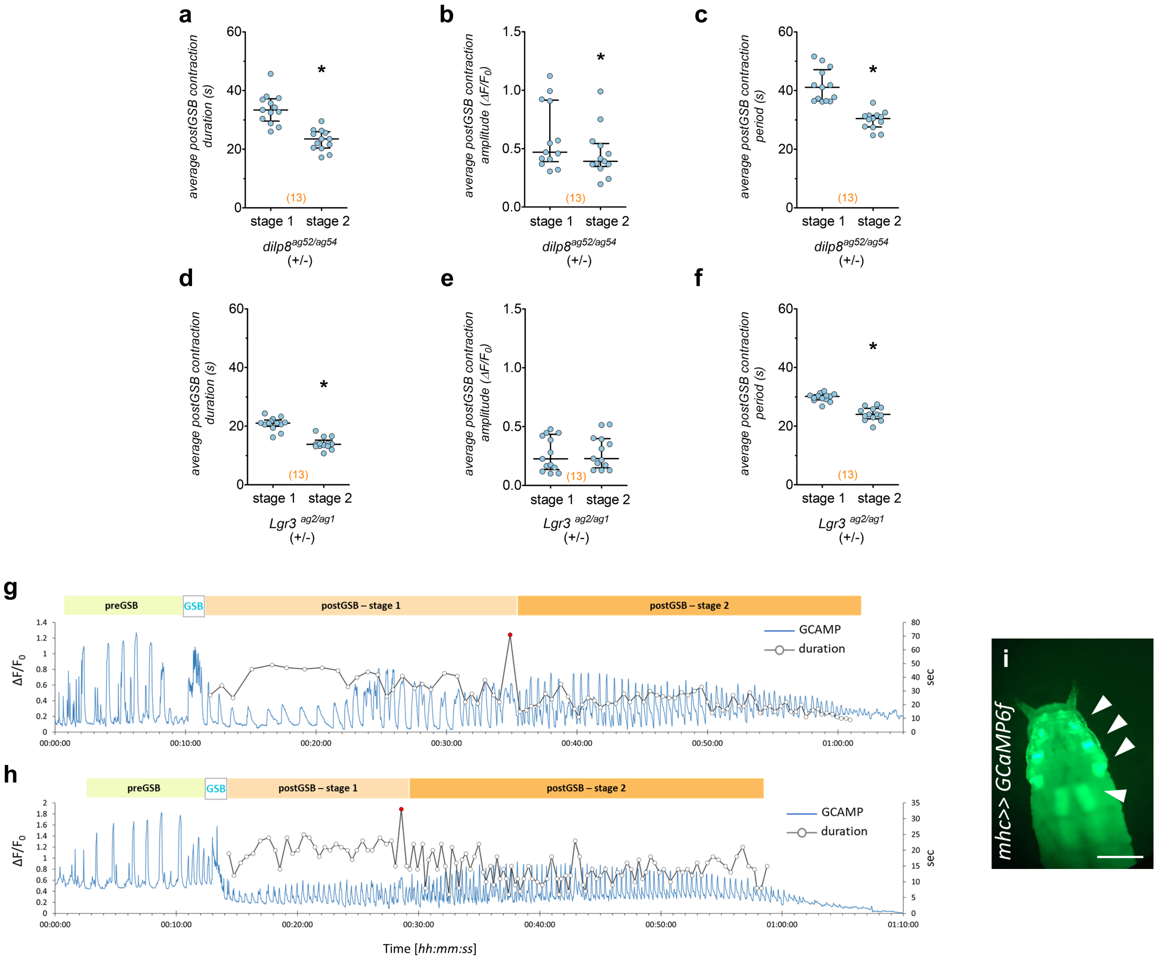

Supplementary Fig. 6: Post-GSB stages and operculum formation program

a Dot plots of average duration, **b** amplitude, and **c** period of post-GSB contractions according to stages in WT animals of the *dilp8*$^{ag52/ag54}$ *(+/-)* genetic background. **d** Dot plots of average duration, **e** amplitude, and **f** period of post-GSB contractions according to stages in WT animals of the *Lgr3*$^{ag2/ag1}$ *(+/-)* genetic background. **g, h** mhc>>GCaMP-fluorescence profiles (blue line) of the pupariation motor program (PMP) of two WT animals indicating the durations of pre-GSB (light green box), GSB (white box and blue font), and post-GSB stages 1 and 2 (light and dark orange boxes, respectively). The duration of each post-GSB peak in seconds (sec) is depicted by the dark line and each white circle corresponds to one contraction. The limit between post-GSB stages 1 and 2 is marked by a contraction that is more prolonged in comparison with the neighboring contractions (marked by the red circle). **i** Dissection microscope photo taken under blue light of the mhc>>GCaMP-fluorescence pattern in a WT animal during operculum formation stage, which probably corresponds to late post-GSB-2. Representative image of >10 monitored animals. Statistics (full details in Supplementary Table 2): **a-f** Dots: average per larva. Horizontal bar, median. Error bars: 25-75% percentiles. **a, c-f** Paired Student's t test**. b** Wilcoxon signed Rank test. *P<0.001. (N) number of animals (orange). $\Delta F/F0 = (F-F0)/F0$, being F0 the minimum value of each trace. Scale bar, 500 µm.

Supplementary Fig. 7.

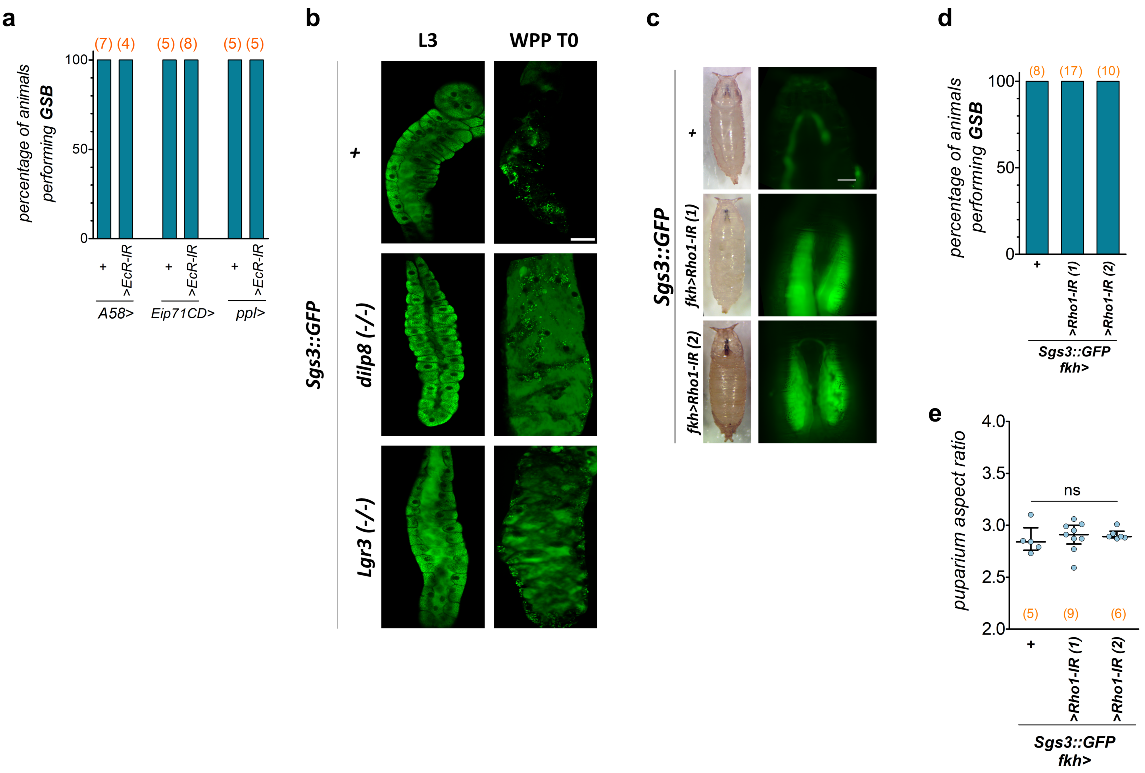

Supplementary Fig. 7: Effects of epidermal *EcR* activity, whole-body *dilp8* and *Lgr3* activity, and glue expulsion activity on puparium morphology and GSB.

a Percentage of animals that perform GSB upon *EcR* knockdown in the epidermis (*A58>EcR-IR* and *Eip7CD>EcR-IR*) or in the fat body (*ppl>EcR-IR*). *A58>/+, Eip7CD>/+,* and *ppl>/+* controls are the same as in Fig. 5i. **b** Confocal sections of dissected salivary glands of the depicted genotypes at L3 (wandering) and WPP T0 (pupariation) stage. Representative images of glands dissected from at least 3 animals per genotype. **c** Photos of WPP T0 puparia and Sgs3::GFP (green) showing that knockdown of *Rho1* using two different RNAi constructs (*>Rho1-IR(1)* and *>Rho1-IR(2)*) in the salivary gland (using *fkh-GAL4, fkh>*) impedes glue expulsion (representative images of at least 5 animals per genotype), but does not **d** affect GSB (percentage of pupa performing GSB) or **e** puparium aspect ratio (AR), depicted by dot plots. Statistics (full details in Supplementary Table 2): **a, d** Binomial test with Bonferroni corrections. **e** Dots: one animal. Horizontal bar, median. Error bars: 25-75% percentiles. ANOVA. ns, not-significant, *P*>0.05. **b** **P*<0.05. (N) number of animals (orange). **b, c** Scale bars, 30 µm and 200 µm, respectively.

Supplementary Fig. 8.

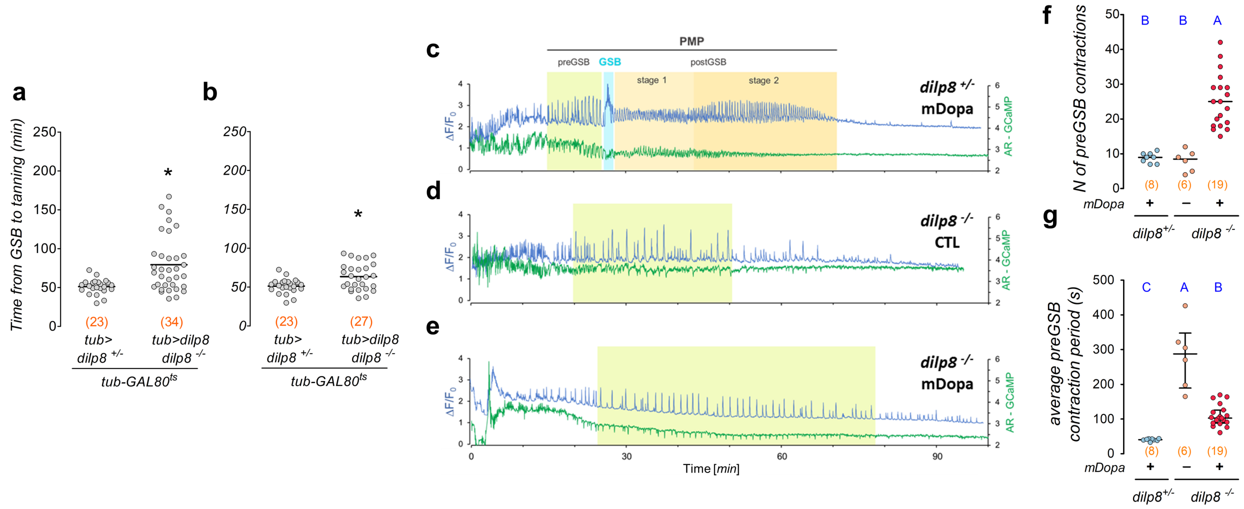

Supplementary Fig. 8: Pupariation progression requires coupling of morphogenetic and neuromotor subprograms by the Dilp8-Lgr3 pathway.

a Dot plots of time from GSB to tanning following removal of animals with double GSB or **b** animals with time >100 min. Expression of *tub>dilp8* after midthird instar transition delays tanning. **c-e** mhc>>GCaMP (blue) and aspect ratio (AR-GCaMP, green) fluctuations in WT animals treated with α-methyldopa (**c**) and *dilp8* mutants treated with vehicle (**d**) or α-methyldopa (**e**). Treatment with α-methyldopa does not overtly alters the PMP of WT animals, nor rescues the defective PMP of mutant animals, which only show pre-GSB-like contractions, never transitioning to GSB. **f** number and **g** average period of pre-GSB contractions. Treatment with α-methyldopa increases the number (**f**) and decreases the average period (**g**) of pre-GSB contractions in *dilp8* mutants. Statistics (full details in Supplementary Table 2): **a, b,** Dots: one animal. Horizontal bar, average. Error bars: 25-75%. Mann-Whitney Rank sum test. *P<0.05. **f, g.** Dots: one animal. Horizontal bar, median. Error bars: 25-75%. Dunn's test. Same blue letters, P>0.05. (N) number of animals (orange).

Supplementary Fig. 9.

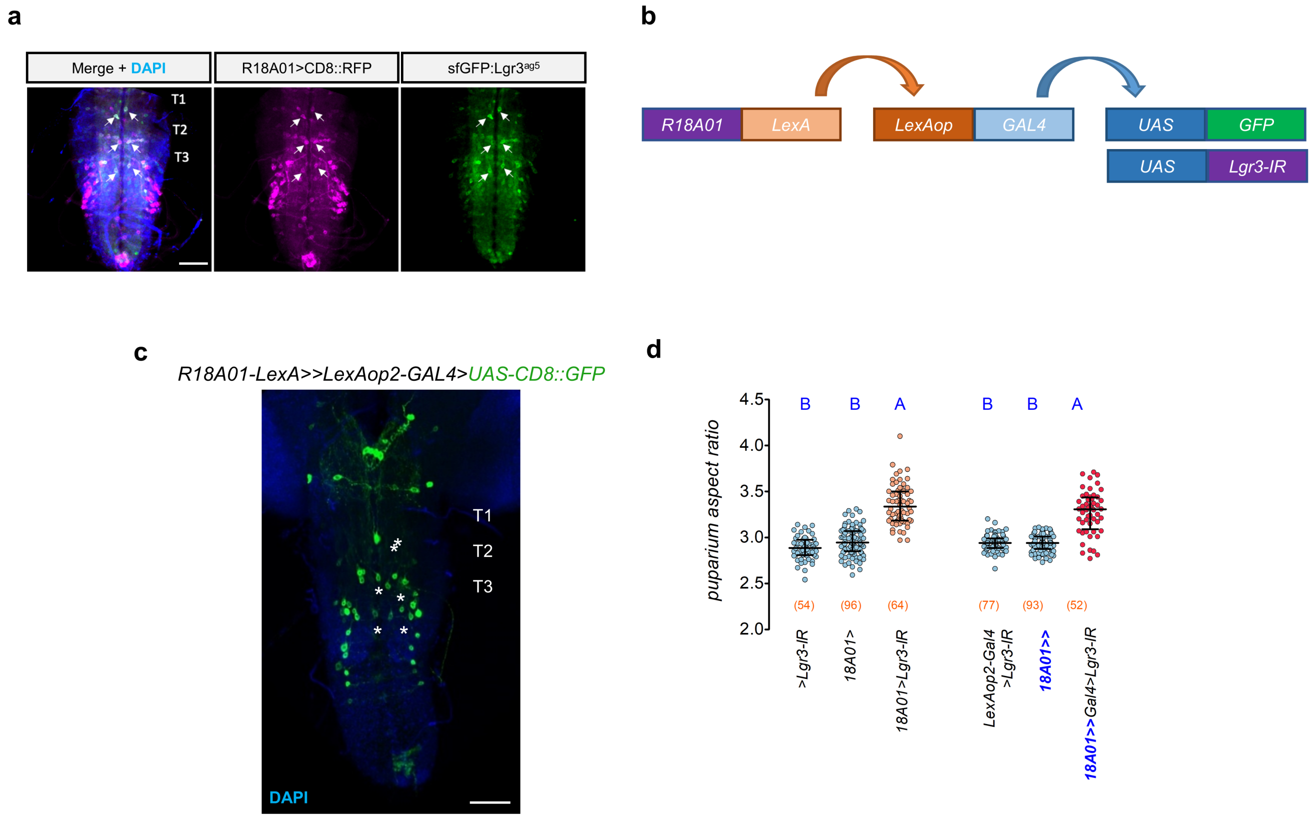

Supplementary Fig. 9: *R18A01* expression pattern and validation of *R18A01-LexA* tools.

a Projections of confocal sections of a dissected white prepupa T0 CNS. Six thoracic (6VNC) interneurons (white arrows) co-express *R18A01>CD8::RFP* (magenta) and *sfGFP::Lgr3^{ag5}* (anti-GFP, green). DAPI, blue. **b** Strategy for testing whether or not the *R18A01-LexA (R18A01>>)* line drives expression in the same *Lgr3*-sensitive cells that are important for pupariation behavior control as the *R18A01-GAL4* (*R18A01>*) line. *R18A01>>* drives expression of LexA which drives expression of GAL4 (*LexAop-GAL4*, *13xLexAop2-GAL4v-VP48*), which then drives expression of either *UAS-Lgr3-IR* or *UAS-CD8::GFP*. This is critical to generate a functional intersection, such as the *R18A01∩R48H10* intersectional genetics system. **c** Projections of confocal sections of a dissected white prepupa T0 CNS. *R18A01>>CD8::GFP* is expressed in the same 6VNC cells (asterisks) as *R18A01>*. T1-3, thoracic segments. **d** Dot plots of puparium aspect ratio (AR). *Lgr3* knockdown under the control of *R18A01>* or *R18A01>>* increases puparium AR (Dots: one animal). Statistics (full details in Supplementary Table 2): **d** Horizontal bar, median. Error bars, 25-75%. Same blue letters, *P*>0.05. Dunn's test. (N) Number of animals (orange). Scale bars, 50 µm.

Supplementary Fig. 10.

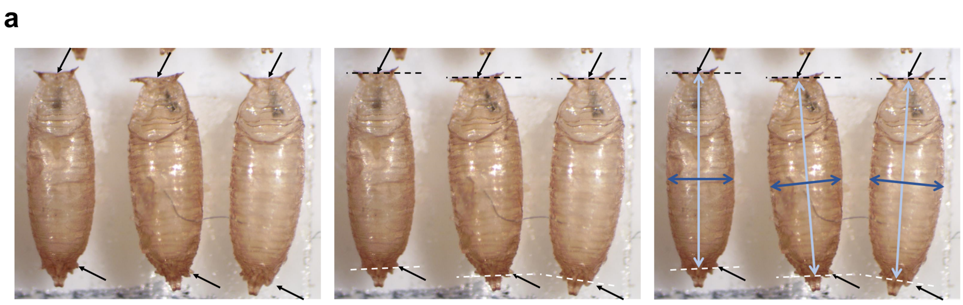

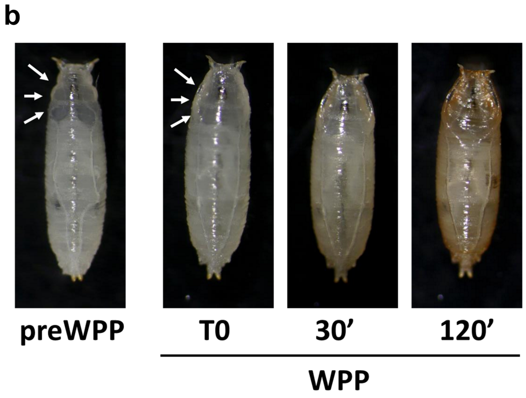

Supplementary Fig. 10: Criteria for puparium aspect ratio and pupa staging

a Measurement of the AR. The black arrows point to the anterior edge and the most anterior anal papillae that were taken as reference to measure the length of the pupa (light blue arrows). A perpendicular line drawn in the widest region of the pupae was used to measure the width. Aspect ratio was calculated as Length/Width. **b** Morphological features of a white prepupa (WPP = T0). A larva is considered to have reached the WPP stage when the operculum first becomes evident, the most anterior segments flatten in the dorso-ventral direction, and their edges thicken and lose the wiggly appearance (arrows). preWPP = animals that have started pupariation, but have not yet reached the WPP stage. Detectable tanning at WPP occurs ~30 min after T0.

Supplementary Fig. 11.

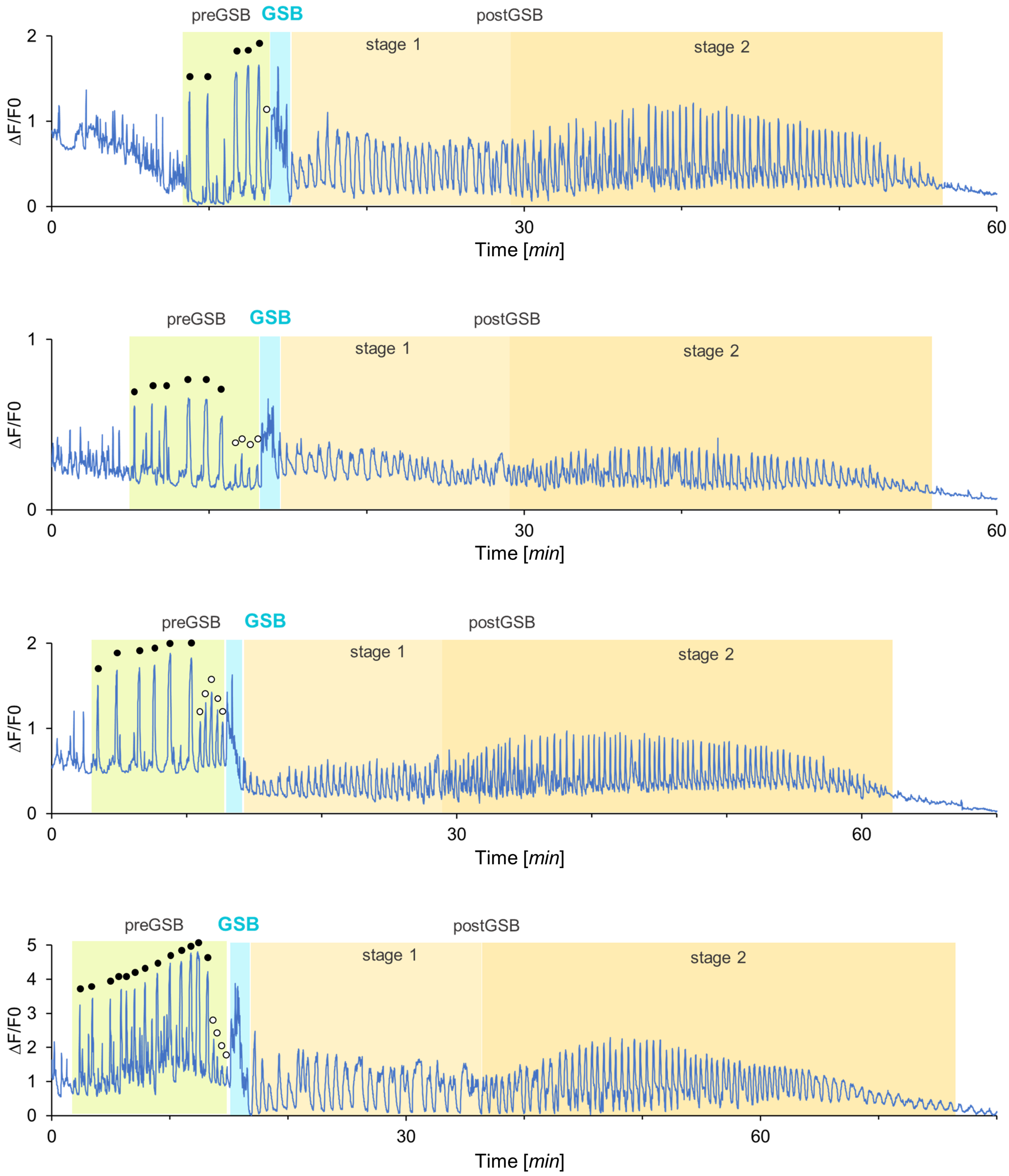

Supplementary Fig. 11: Examples of mhc>>GCaMP fluorescence traces of WT animals in which the different components of the pupariation motor program (PMP) are depicted.

The PMP begins once the larva has stopped wandering (see Fig. 4). Pre-GSB contractions are marked with a black circle. White circles indicate small fluctuations of mhc>>GCaMP signal intensity that occur between the last strong pre-GSB contraction and GSB and are the result of milder and shorter body contractions. $\Delta F/F0 = (F-F0)/F0$, being F0 the minimum value of each trace. Genotypes: Top three, *Lgr3*$^{ag2/ag1}$ *(+/-).* Bottom, *dilp8*$^{ag52/ag54}$ *(+/-).*

Supplementary Fig. 12.

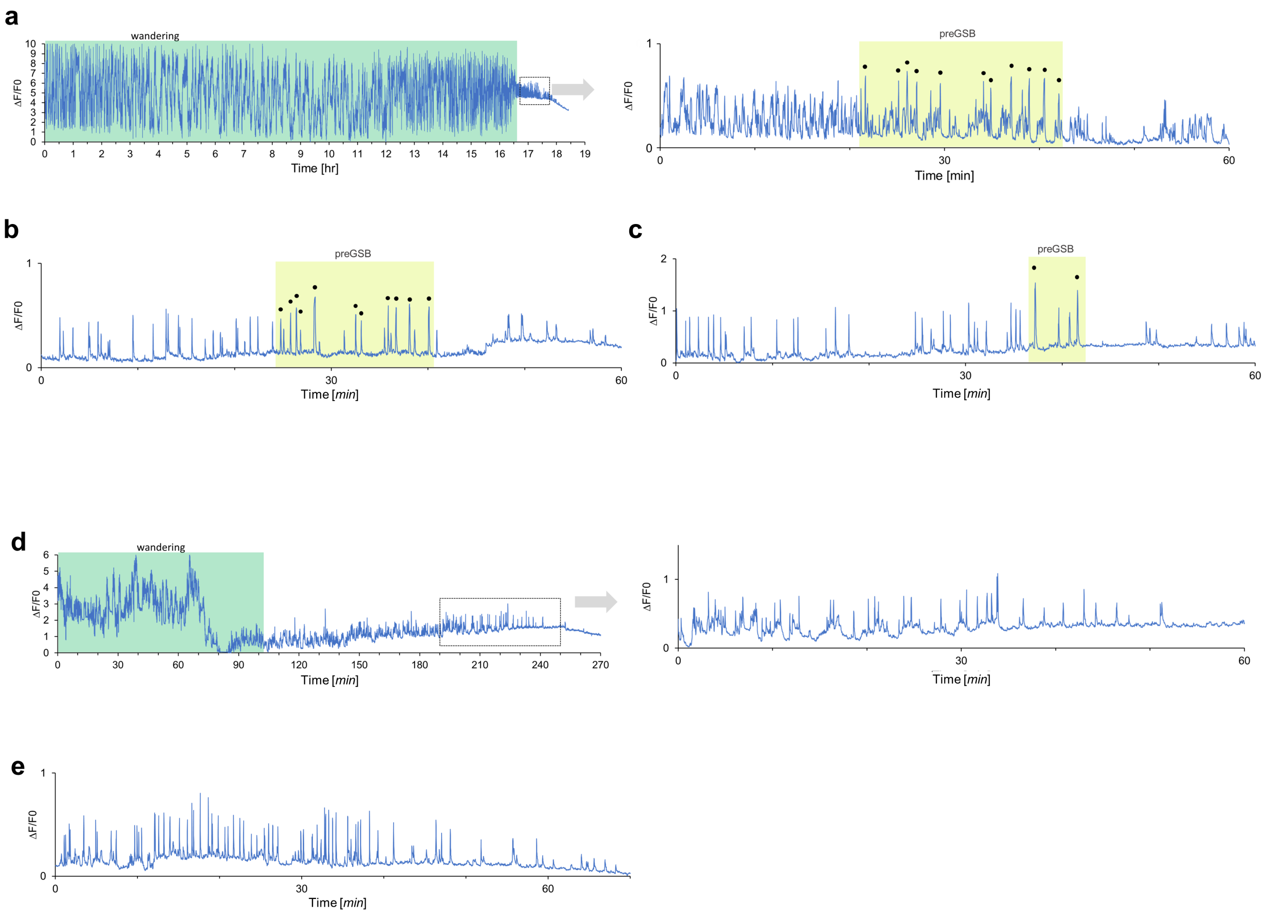

Supplementary Fig. 12: Examples of mhc>>GCaMP-fluorescence traces of *Lgr3* mutant animals.

a Similarly to WT animals, *Lgr3*$^{ag1/ag1}$ *(-/-)* mutant larvae wander along the arena for a variable time, sometimes as long as 16 h, until they stop and select a place to pupariate. Next, most animals perform pre-GSB-like contractions, but fail to progress further in the pupariation motor program. The graph on the right is an expansion of the region delimited by the dashed-line box, in which the peaks in total mhc>>GCaMP-fluorescence signals that result from whole body contractions have been indicated with a black circle. **b, c** mhc>>GCaMP-fluorescence traces of two different mutant larvae that perform pre-GSB-like contractions, representative of the variability observed among individuals. **d, e** Examples of mutant larvae with no detectable whole-body preGSB contraction. $\Delta F/F0 = (F-F0)/F0$, being F0 the minimum value of each trace.

Supplementary Table 1.

Supplementary Table 1: List of primers used in this study.

Gene/target	primer name	5'-3' sequence
Dilp8-gRNA	#200_DILP8-GuideRNA_1_F	CTTCGCACTGGTTTAGACAGCAGT
Dilp8-gRNA	#201_DILP8-GuideRNA_1_R	AAACACTGCTGTCTAAACCAGTGC
Dilp8-gRNA	#107_dilp8_salto_exon2_R	CAGTTGCATATGTGCCGCTGGA
Dilp8-gRNA	#681_DILP8-GuideRNA_1_F-ALT	TGCAGCACTGGTTTAGACAGCAGT
LHV2	D-TOPO_LHV2_F	CACCAAGCCTCCTGAAAGATG
LHV2	D-TOPO_LHV2_R	AATGTATCTTATCATGTCTAGAT
D. mel rp49	AL37_RP49-1S	TGTCCTTCCAGCTTCAAGATGACCATC
D. mel rp49	AL38_RP49-1AS	CTTGGGCTTGCGCCATTTGTG
D. mel dilp8	AL29_CG14059 qPCR-L	CGACAGAAGGTCCATCGAGT
D. mel dilp8	AL30_CG14059 qPCR-R	GATGCTTGTTGTGCGTTTTG
D. mel pale	TH_forward	TTCGGAGGCGGCATTG
D. mel pale	TH_reverse	ACAGCCGACCAAGAACGATT
D. mel dib	AL163_dib_F	GTGACCAAGGAGTTCATTAGATTTC
D. mel dib	AL164_dib_R	CCAAAGGTAAGCAAACAGGTTAAT
D. mel phm	AL165_phm_F	TAAAGGCCTTGGGCATGA
D. mel phm	AL166_phm_R	TTTGCCTCAGTATCGAAAAGC
D. mel $E74B^{121}$	AL1013_E74B_245bp_Fw	ATGGGCAGCAGGCTAAGACTCAG
D. mel $E74B^{121}$	AL1014_E74B_245bp_Rv	TACGGATTCAGACTCCTCTTCATC
C. cap RpL32 (rp49)	41_Ccap_RpL32_F	TAACAGAGTACGTCGTCGTTTCA
C. cap RpL32 (rp49)	42_Ccap_RpL32_R	GAATTTCTTGAAGCCAGTTGGT
C. cap ilp8 (cilp8)	47_Ccap ilp8_p1_F	TGCAAGATGAGGGTAGAGAGAAG
C. cap ilp8 (cilp8)	48_Ccap ilp8_p1_R	CTTGACCCACTTTCAGATAACCA
C. cap cilp8 in situ	#654 cilp8_probe_fwd	TGAGAACACTATTCCTTACATTCTTC
C. cap cilp8 in situ	#655 cilp8_probe_rev	GAAATCCTCTTCACATTTGTTGT

Supplementary Table 2.

Supplementary Table 2. Statistical analyses information for each figure.

Figure		tested genotypes	test	statistic -- degrees of freedom	P value	multiple comparisons test	P
1	B	all	Kruskal-Wallis	H = 209.766 -- 2 df	<0.001	Dunn	<0.05
	D	all	Kruskal-Wallis	H = 188.484 -- 2 df	<0.001	Dunn	<0.05
	F	all	Kruskal-Wallis	H = 289.936 -- 5 df	<0.001	Dunn	<0.05
	G	1-2-3	Kruskal-Wallis	H = 55.086 -- 2 df	<0.001	Dunn	<0.05
		1-4-5	Kruskal-Wallis	H = 12.227 -- 2 df	0.002	Dunn	<0.05
		1-6-7	Kruskal-Wallis	H = 2.836 -- 2 df	0,242	---	---
		1-8-9	One way ANOVA	F=73.066 -- 2 df	<0.001	Tukey Test	<0.05
		1-10-11	One way ANOVA	F= 0.425 -- 2 df	0.655	---	---
		1-12-13	Kruskal-Wallis	H = 83.975 -- 2 df	<0.001	Dunn	<0.05
		1-14-15	One way ANOVA	F=10.649 -- 2 df	<0.001	Tukey Test	<0.05
		1-16-17	Kruskal-Wallis	H = 125.973 -- 2 df	<0.001	Dunn	<0.05
	J	1-2	Kruskal-Wallis	H = 209.528 -- 1 df	<0.001	Dunn	<0.05
		3-4-5	Kruskal-Wallis	H = 279.000 -- 2 df	<0.001	Dunn	<0.05
		6-7-8	Kruskal-Wallis	H = 379.820 -- 2 df	<0.001	Dunn	<0.05
		9-10-11	Kruskal-Wallis	H = 216.408 -- 2 df	<0.001	Dunn	<0.05
		12-13-14	Kruskal-Wallis	H = 378.533 -- 2 df	<0.001	Dunn	<0.05
		15-16-17	Kruskal-Wallis	H = 8.115 -- 2 df	0.017	Dunn	<0.05
	K	1-2-3	Kruskal-Wallis	H = 65.159 -- 2 df	<0.001	Dunn	<0.05
		1-4-5	Kruskal-Wallis	H = 12.651 -- 2 df	0.002	Dunn	<0.05
		1-6-7	Kruskal-Wallis	H = 32.645 -- 2 df	<0.001	Dunn	<0.05
		1-8-9	Kruskal-Wallis	H = 141.161 -- 2 df	<0.001	Dunn	<0.05
		1-10-11	Kruskal-Wallis	H = 12.219 -- 2 df	0.002	Dunn	<0.05
	L	all	Kruskal-Wallis	H = 124.084 -- 4 df	<0.001	Dunn	<0.05

2	A	all	One Way ANOVA	F = 31.986 -- 6 df	<0.001	Student-Newman-Keuls	<0.05
	B	all	One Way ANOVA	F = 74.623 -- 6 df	<0.001	Student-Newman-Keuls	<0.05
	C	dilp8 3 hr	One tailed T-test	t= -1.984 -- 4 df	0.0591	---	---
		dilp8 6 hr	One tailed T-test	t= -7.204 -- 4 df	0.00197	---	---
	D	pale 3 hr	One tailed T-test	t= -2.657 -- 4 df	0.0565	---	---
		pale 6 hr	One tailed T-test	t= -0.557 -- 4 df	0.607	---	---

	F	all	Kruskal-Wallis	H = 198.795 -- 5 df	<0.001	Dunn	<0.05
	G	all	One Way ANOVA	F = 54.159 -- 13 df	<0.001	Holm-Sidak	<0.05
	H	all	One Way ANOVA	F = 19.379 -- 8 df	<0.001	Student-Newman-Keuls	<0.05
	I	all	One Way ANOVA	F = 77.619 -- 5 df	<0.001	Student-Newman-Keuls	<0.05

3	B	all	Kruskal-Wallis	H = 19.480 -- 3 df	<0.001	Dunn	<0.05
3	C	all	Kruskal-Wallis	H = 131.691 -- 7 df	<0.001	Dunn	<0.05
3	E	all	Kruskal-Wallis	H = 169.29 -- 9 df	<0.0001	Conover	<0.05

4	E	all	Mann-Whitney test	U = 111.000	0.763	---	---
4	F	all	One tailed T-test	t= -7.342 -- 29 df	2.17E-08	---	---
4	G	all	Mann-Whitney test	U = 67.000	0.041	---	---
4	H	all	Mann-Whitney test	U = 17.000	<0.001	---	---
4	L	all	Kruskal-Wallis	H = 76.919 -- 3 df	<0.001	Dunn	<0.05

5	A		Binomial test	the (one-tailed) probability of exactly, or fewer/more than observed value is	
			Z	more	fewer
		dilp8 +/+	-1.028771	0.87780914	
		dilp8 +/-	-1.629326	0.93223012	
		dilp8 -/-	-101.72487		<0.000001
		Lgr3 +/+	-1.370096	0.91372489	
		Lgr3 +/-	-1.555775	0.92756897	
		Lgr3 -/-	-90.328271		<0.000001
	C	no statistical analysis was performed			
	D	no statistical analysis was performed			
	E		Binomial test	the (one-tailed) probability of exactly, or fewer/more than observed value is	
			Z	more	fewer
		dilp8 +/-	-1.629326	0.93223012	
		dilp8 -/-	-63.09828		<0.000001
		Lgr3 +/-	-1.555775	0.92756897	
		Lgr3 -/-	-69.115917		<0.000001
	F		Binomial test	the (one-tailed) probability of exactly, or fewer/more than observed value is	
			Z	more	fewer
		Lgr3-IR/+	-0.905964	0.86038419	

Panel	Genotype	Construct	Temperature	Z / Test	more	fewer
	18A01-Gal4/+			-39.52062		<0.000001
	18A01>Lgr3-IR			- 53.005694		<0.000001
	19B09-Gal4/+			-1.42701	0.91831647	
	19B09>Lgr3-IR			-1.178845	0.89558699	
	18A01-Gal4/+			Fisher Exact test	p = 0.3041	
	18A01>Lgr3-IR				alpha = 0.05	
G				Binomial test	the (one-tailed) probability of exactly, or fewer/more than observed value is	
				Z	more	fewer
	Lgr3 -/-	UAS-Lgr3/+		0.905964	0 .182477	
	Lgr3 -/-	18A01-Gal4/+		0.852133	0.19707	
	Lgr3 -/-	18A01>Lgr3		27.432272	<0.000001	
H			temperature	Binomial test	the (one-tailed) probability of exactly, or fewer/more than observed value is	
				Z	more	fewer
	dilp8 +/-		18	0.473684	0.317863	
	dilp8 +/-		30	0.586939	0.278622	
	dilp8-/-	+	18	0.802376		0.84330514
	dilp8-/-	+	30	0.905964		0 .86038419
	dilp8-/-	UAS-dilp8WT	18	0.734198		0.8307187
	dilp8-/-	UAS-dilp8WT	30	5.081527	<0.000001	
	dilp8-/-	UAS-dilp8C150A	18	1.317291		0.90915626
	dilp8-/-	UAS-dilp8C150A	30	1.063322		0.88222024
I				Binomial test	the (one-tailed) probability of exactly, or fewer/more than observed value is	
				Z	more	fewer
	ppl>	+		-3.011702	0.97524875	
	ppl>	>dilp8-IR-TRIP		-3.011702	0.97524875	
	ppl>	>dilp8-IR-TRIP>Dcr2		-3.40263	0.9801495	
	A58>	+		-2.491767	.96552065	
	A58>	>dilp8-IR-TRIP		-1.8008	0.94162281	
	A58>	>dilp8-IR-TRIP>Dcr2		-2.720356	0.97037251	
	Eip71CD>	+		-3.011702	0.97524875	
	Eip71CD>	>dilp8-IR-TRIP		-2.491767	.96552065	
	Eip71CD>	>dilp8-IR-TRIP>Dcr2		-1.710492	0.93691469	
	A58> + Eip71CD>	+		-1.902248	0.94635458	
	A58> + Eip71CD>	>dilp8-IR-TRIP		-1.555775		0.0598810
	A58> + Eip71CD>	>dilp8-IR-TRIP>Dcr2		-5.642657		0.0018798
K				Binomial test	the (one-tailed) probability of exactly, or fewer/more than observed value is	
				Z	more	fewer

		+		-1.710492		0.06308531
		dilp8 -/-		-66.17541		<0.000001
		Lgr3 -/-		-46.820803		<0.000001

Figure		tested genotypes	test	statistic -- degrees of freedom	P value	multiple comparisons test	P
6	A	all	One Way ANOVA	F = 31.385 -- 3 df	<0.001	Student-Newman-Keuls	<0.05
	B	all	Mann-Whitney test	U = 206.500	<0.001	---	---
	E	all	One Way ANOVA	F = 174.722 -- 3 df	<0.001	Student-Newman-Keuls	<0.05
	F	all	Kruskal-Wallis	H = 61.988 -- 3 df	<0.001	Dunn	<0.05
	H	all	One Way ANOVA	F = 10.818 -- 2 df	<0.001	Student-Newman-Keuls	<0.05
	J	all	Mann-Whitney test	U = 0.000	0.001	---	---
	G			mDOPA	Binomial test	the (one-tailed) probability of exactly, or fewer/more than observed value is	
					Z	more	fewer
		dilp8 (+/-)		YES	-2.305772	0.96069304	
		dilp8 (-/-)		NO	-34.643596		<0.000001
				YES	-74.650739		<0.000001
		Lgr3 (+/-)		YES	-2.491767	0.96552065	
		Lgr3 (-/-)		YES	-28.373407		<0.000001

Figure		tested genotypes	test	statistic -- degrees of freedom	P value	multiple comparisons test	P
7	A	all	One Way ANOVA	F = 57.923 -- 3 df	<0.001	Holm-Sidak	<0.01
	H	all	Kruskal-Wallis	H = 166.698 -- 4 df	<0.001	Dunn	<0.05
	J	all	Kruskal-Wallis	H = 85.460 -- 2 df	<0.001	Dunn	<0.05
	K	all	Kruskal-Wallis	H = 162.946 -- 4 df	<0.001	Dunn	<0.05
	N	dib	One Way ANOVA	F = 2.677 -- 2 df	0.148	---	---
		phm	One Way ANOVA	F = 0.916 -- 2 df	0.449	---	---
		E74B	One Way ANOVA	F = 0.142 -- 2 df	0.871	---	---

			Binomial test	the (one-tailed) probability of exactly, or fewer/more than observed value is	
			Z	more	fewer
	B	UAS-Lgr3-IR/+	-0.905964	0.86038419	
		R48H10>+	-1.222055	0.90008743	
		R48H10>Lgr3-IR	-42.367737		<0.000001
	I		Binomial test	the (one-tailed) probability of exactly, or fewer/more than observed value is	
			Z	more	fewer
		+	-2.150273	0.99912077	
		R18A01-LexA	-64.655117		<0.000001
		R48H10-Gal4	-12.537715		<0.000001
		R18A01-LexA + R48H10-Gal4	-69.115917		<0.000001

Figure		tested genotypes	test	statistic -- degrees of freedom	P value	multiple comparisons test	P
S1	C	all	Kruskal-Wallis	H = 17.612 -- 2 df	<0.001	Dunn	<0.05
	E	all	One way ANOVA	F = 12.332 -- 2 df	<0.001	Student-Newman-Keuls	<0.05
	G	all	Kruskal-Wallis	H = 67.995 -- 3 df	<0.001	Dunn	<0.05
	I	all	One tailed T-test	t= -10.569 -- 99 df	3.16E-15	---	---

S3	B	all	Kruskal-Wallis	H = 103.022 -- 6 df	<0.001	Dunn	<0.05
	D	all	Kruskal-Wallis	H = 73.115 -- 2 df	<0.001	Dunn	<0.05
	I	all	One way ANOVA	F = 49.16 -- 8 df	<0.0001	Bonferroni Multiple Comparison Test on selected genotypes	<0.05

			Binomial test	the (one-tailed) probability of exactly, or fewer/more than observed value is	
S3	F		Z	more	fewer
		dilp8[ag52]	-0.027875		0.488881
		dilp8[ag51]	46.580187	<0.000001	
		dilp8[ag50]	19.128888	<0.000001	
		dilp8[ag55]	13.534546	<0.000001	
		dilp8[ag54]	27.927617	<0.000001	
		Lgr3 ag2	-0.75288		0.225761
		Lgr3 ag1	49.538978	<0.000001	
	G	dilp8[ag52]	-1.84807		0.032296
		dilp8[ag51]	49.347385	<0.000001	

		dilp8[ag50]	39.879882	<0.000001	
		dilp8[ag55]	*25.52312*	<0.000001	
		dilp8[ag54]	27.991357	<0.000001	
		Lgr3 ag2	-1.38942		0.082353
		Lgr3 ag1	38.71932	<0.000001	
	H	Lgr3IR/+	-1.707741		0.043842
		tub>	0.089131		0.464489
		tub>Lgr3IR	23.541748	<0.000001	
		57C10>	0.214047		0.415255
		57C10>Lgr3IR	26.531754	<0.000001	

Figure		tested genotypes	test	statistic -- degrees of freedom	P value	multiple comparisons test	P
S4	C	all	Mann-Whitney test	U = 114.000	0.858	---	---
	D	all	Mann-Whitney test	U = 73.000	0.26	---	---
	E	all	One tailed T-test	t= -1.622 -- 28 df	0.0581	---	---
	F	all	One tailed T-test	t= -7.570 -- 28 df	1.51E-08	---	---
	G	all	Mann-Whitney test	U = 73.000	0.106	---	---
	H	all	Mann-Whitney test	U = 12.000	<0.001	---	---
	L	all	Kruskal-Wallis	H = 69.883 -- 3 df	<0.001	Dunn	<0.05

S6	A	all	Paired t-test (one tailed)	t= 12.664 -- 12 df	1.32E-08	---	---
	B	all	Wilcoxon Signed Ranked Test	Z= -3.110	<0.001	---	---
	C	all	Paired t-test (one tailed)	t= 8.535 -- 12 df	9.64E-07	---	---
	D	all	Paired t-test (one tailed)	t= 14.438 -- 12 df	3.01E-09	---	---
	E	all	Paired t-test (one tailed)	t= -0.756 -- 12 df	0.232	---	---
	F	all	Paired t-test (one tailed)	t= 8.907 -- 12 df	6.16E-07	---	---

S7	A				Binomial test	the (one-tailed) probability of exactly, or fewer/more than observed value is	
					Z	more	fewer
		A58>	+		-2.491767	0.96552065	
			EcR-IR		-3.40263	0.9801495	
		Eip71CD>	+		-3.011702	0.97524875	
			EcR-IR		-2.305772	0.96069304	
		ppl>	+		-3.011702	0.97524875	
			EcR-IR		-3.011702	0.97524875	
	D	Sgs3::GFP fkh> +			-2.305772	0.96069304	
		Sgs3::GFP fkh> Rho1-IR(1)			-1.42701	0.91831647	
		Sgs3::GFP fkh> Rho1-IR(2)			-2.017511	0.95111013	
	E	tested genotypes	test	statistic	P value	multiple comparisons test	P
		all	One way ANOVA	F = 0.231 -- 2 df	0.796	---	---

S8	A	all	Mann-Whitney test	U = 193.500	0.001	---	---
	B	all	Mann-Whitney test	U = 193.500	0.023	---	---
	F	all	Kruskal-Wallis	H = 23.576 -- 2 df	<0.001	Dunn	<0.05
	G	all	Kruskal-Wallis	H = 25.002 -- 2 df	<0.001	Dunn	<0.05

S9	D	all	Kruskal-Wallis	H = 200.249 -- 5 df	<0.001	Dunn	<0.05
