# Supplementary material for: The steroid-hormone ecdysone coordinates parallel pupariation neuromotor and morphogenetic subprograms via epidermis-to-neuron Dilp8-Lgr3 signal induction
Source: Nat Commun. 2021 Jun 7;12:3328. doi: 10.1038/s41467-021-23218-5 (PMC8184853; doi:10.1038/s41467-021-23218-5)
Supplement: Supplementary file 13 — Reporting Summary [file 41467_2021_23218_MOESM13_ESM.pdf]

nature research

Corresponding author(s): Alisson M. Gontijo and Andres Garelli

Last updated by author(s): Mar 5, 2021

Reporting Summary

Nature Research wishes to improve the reproducibility of the work that we publish. This form provides structure for consistency and transparency in reporting. For further information on Nature Research policies, see our Editorial Policies and the Editorial Policy Checklist.

Statistics

For all statistical analyses, confirm that the following items are present in the figure legend, table legend, main text, or Methods section.

n/a	Confirmed	
☐	☒	The exact sample size (*n*) for each experimental group/condition, given as a discrete number and unit of measurement
☐	☒	A statement on whether measurements were taken from distinct samples or whether the same sample was measured repeatedly
☐	☒	The statistical test(s) used AND whether they are one- or two-sided<br>*Only common tests should be described solely by name; describe more complex techniques in the Methods section.*
☒	☐	A description of all covariates tested
☐	☒	A description of any assumptions or corrections, such as tests of normality and adjustment for multiple comparisons
☐	☒	A full description of the statistical parameters including central tendency (e.g. means) or other basic estimates (e.g. regression coefficient) AND variation (e.g. standard deviation) or associated estimates of uncertainty (e.g. confidence intervals)
☐	☒	For null hypothesis testing, the test statistic (e.g. *F*, *t*, *r*) with confidence intervals, effect sizes, degrees of freedom and *P* value noted<br>*Give P values as exact values whenever suitable.*
☒	☐	For Bayesian analysis, information on the choice of priors and Markov chain Monte Carlo settings
☒	☐	For hierarchical and complex designs, identification of the appropriate level for tests and full reporting of outcomes
☒	☐	Estimates of effect sizes (e.g. Cohen's *d*, Pearson's *r*), indicating how they were calculated

Our web collection on statistics for biologists contains articles on many of the points above.

Software and code

Policy information about availability of computer code

Data collection	https://github.com/AndresGarelli/ImageJ-Larva-Tracking-Tool<br>https://github.com/AndresGarelli/FlyPi-Pupariation
Data analysis	Microsoft Excel (version 16.41), FIJI (ImageJ Version 2.0.0-rc-69/1.52p), SigmaPlot package version 12.0, https://www.socscistatistics.com/, and other resources provided in data collection. Amscope v3.7.7934.

For manuscripts utilizing custom algorithms or software that are central to the research but not yet described in published literature, software must be made available to editors and reviewers. We strongly encourage code deposition in a community repository (e.g. GitHub). See the Nature Research guidelines for submitting code & software for further information.

Data

Policy information about availability of data

All manuscripts must include a data availability statement. This statement should provide the following information, where applicable:
- Accession codes, unique identifiers, or web links for publicly available datasets
- A list of figures that have associated raw data
- A description of any restrictions on data availability

Data Availability
All relevant data are available from the authors. Source data are provided with this paper. Data used in Supplementary Figs. 2a,d were obtained from microarray data(Ref.55) deposited in the National Center for Biotechnology Information Gene Expression Omnibus website, with accession numbers as follows: GSE3057 for Supplementary Figs. 2a,d and GSE3069 for Supplmentary Figs. 2c,f. Data used in Supplementary Figs. 2b,e were obtained from microarray data(Ref.54) available from https://doi.org/10.1016/S1534-5807(03)00192-8.

Field-specific reporting

Please select the one below that is the best fit for your research. If you are not sure, read the appropriate sections before making your selection.

☒ Life sciences ☐ Behavioural & social sciences ☐ Ecological, evolutionary & environmental sciences

For a reference copy of the document with all sections, see nature.com/documents/nr-reporting-summary-flat.pdf

Life sciences study design

All studies must disclose on these points even when the disclosure is negative.

Sample size	Ns are described in each figure for each genotype. For developmental time experiments, we measured at least 80 animals per genotype, in accordance with published data (Garelli et al, Science 2012; Garelli et al, Nat. Commun. 2015; Vallejo et al, Science 2012). Mutation of dilp8 and Lgr3 induces an overt change in pupal shape evident with the naked eye. A minimum sample of 20 animals was found to be sufficient to represent the range of aspect ratio values of wild type and mutant populations. For most experiments, at least 40 animals were measured, considering each animal an experimental point, following previous reports (Guan et al., PNAS, 2006; Tajiri et al., PLOS Genetics, 2007). In methodologically complex experiments in which larvae were shifted from 18ºC to 29ºC at specific developmental timepoints, smaller samples were collected, ranging from 19 to 41 individuals. For other behavior analyses we aimed for >10 animals, even though in some cases we stopped at less due to a cost-benefit analyses (e.g., when confirming similar gross phenotypes in different backgrounds). For in vitro treatments and quantitative PCR we performed at least 3 biological repeats.
Data exclusions	No data points were excluded in this work. All data points were represented in the figures and used in statistical analyses.
Replication	The findings have been replicated with the same technique and/or independently with different techniques or genotypes. For the developmental, morphological, and behavioral experiments, the animal is the experimental point, so >10 replications were typically performed, and observations were confirmed at least in 3 independent samples for whole animal long-term calcium imaging and expression/immunofluorescnce pattern analyses. In vitro experiments were reproduced at least 3 times.
Randomization	No specific algorithm of randomization was used in this study, but care was taken to avoid types of selection biases (specifically collection order and time), by randomly selecting and attributing animals/samples to experimental groups in the few experiments where these were collected from the same batch and distributed for specific treatments. Specifically, for quantitative PCR, staged animals were randomly selected from low density cultures. For in vitro treatments, samples from staged animals were randomly assigned to EtOH or 20HE treatment. For developmental time and morphological experiments, animals developed in low density cultures (~30 larvae per vial) and all animals of the relevant genotype within each vial were evaluated.
Blinding	No blinding was performed in this study. Instead we relied upon replication, independent confirmation of results by more than one investigator of the team, independent re-analyses/quantifications, and replication by independent technique and genotypes.

Reporting for specific materials, systems and methods

We require information from authors about some types of materials, experimental systems and methods used in many studies. Here, indicate whether each material, system or method listed is relevant to your study. If you are not sure if a list item applies to your research, read the appropriate section before selecting a response.

Materials & experimental systems

n/a	Involved in the study	
☐	☒	Antibodies
☒	☐	Eukaryotic cell lines
☒	☐	Palaeontology and archaeology
☐	☒	Animals and other organisms
☒	☐	Human research participants
☒	☐	Clinical data
☒	☐	Dual use research of concern

Methods

n/a	Involved in the study	
☒	☐	ChIP-seq
☒	☐	Flow cytometry
☒	☐	MRI-based neuroimaging

Antibodies

Antibodies used	rabbit anti-GFP 1:200 (Life technologies, A11122), mouse anti-Fasciclin III (anti-FasIII)1:50 (Drosophila Studies Hybridoma Bank (DSHB), 7G10]
Validation	The anti-GFP antibody detects GFP variants and sfGFP as shown genetically in Garelli et al., Nat Commun 2015. The anti-FasIII antibody has been previously described Patel et al., Cell (1987) and is widely used by the fly community (commercially available via DSHB).

Animals and other organisms

Policy information about studies involving animals; ARRIVE guidelines recommended for reporting animal research

Laboratory animals	Established Drosophila melanogaster and Drosophila virilis stocks were used. Males and female individuals were used, if not specifically described. The age of the animals was the following: all animals were assayed at larval or pupal stage. Ceratitis capitata culture was kindly provided by Dr. A. Jessup. These are the stocks used in the study:<br><br>Drosophila virilis (15010-1051.118 from The National Drosophila Species Stock Center) was a gift from N. Frankel. All other Drosophila stocks were Drosophila melanogaster. UAS-dilp8 and UAS-dilp8C150A were previously described24. Lgr3ag1, Lgr3ag2, sfGFP::Lgr3ag5, and UAS-Lgr3 were previously described26. tub-dilp8 (Ref. 27) was a gift from M. Dominguez. Feb36-GAL4 (from C. Thummel)102. w; phm-GAL4/TM6Tb, and y w;P0206-GAL4 (Ref. 103) were gifts from C. Mirth. Act88F-GAL4 (from F. Schnorrer)104. ppl-GAL4 (Ref. 105) and dilp8KO (Ref. 40) were gifts from P. Leopold. A58-GAL4 was a gift from M. Galko (Ref. 106). nSyb-GAL4 (III) 107 was a gift from R. Teodoro. UAS–Rho1-IR(1) (VDRC 12734), UAS-Rho1-IR(2) (BL27727 y1 v1; P{y[+t7.7] v[+t1.8]=TRiP.JF02809}attP2), and forkhead-GAL4 (BL78060 w[*]; P{w[+mC]=fkh-GAL4.H}3) were a gift from M. Melani. y1 w67c23; P(CaryP)attP40;; was obtained from the Champalimaud Foundation Injection Facility (a gift from N. Perrimon). UAS-dilp8-IR (v102604)({KK112161}VIE-260B)) and UAS-EcR-IR (w[1118]; P{GD1428}v37059) (Ref. 108) were obtained from the Vienna Drosophila Resource Center (VDRC). vas-int; attP40 (Stock 13-20), full genotype: y w M(eGFP, vas-int, dmRFP)ZH-2A; P{CaryP}attP40 (Ref. 109) was obtained from Fly Facility, Department of Genetics, University of Cambridge.<br><br>The following stocks were obtained from the Bloomington Drosophila Stock Center at Indiana University:<br><br>BL33079 y1 w*; Mi{MIC}Ilp8MI00727<br>BL54591 y1 M{w[+mC]=nos-Cas9.P}ZH-2A w*<br>BL58986 P{ry[+t7.2]=hsFLP}12, y1 w*; P{y[+t7.7] w[+mC]=UAS-Cas9.P2}attP2/TM6B, Tb1<br>BL49275 w1118; P{y[+t7.7] w[+mC]=GMR17G11-GAL4}attP2<br>BL48786 w1118; P{y[+t7.7] w[+mC]=GMR17H01-GAL4}attP2<br>BL48806 w1118; P{y[+t7.7] w[+mC]=GMR18C07-GAL4}attP2<br>BL48791 w1118; P{y[+t7.7] w[+mC]=GMR18A01-GAL4}attP2<br>BL48840 w1118; P{y[+t7,7] w[+mC]=GMR19B09-GAL4}attP2<br>BL39171 w1118; P{y[+t7.7] w[+mC]=GMR57C10-GAL4}attP2<br>BL50395 w1118; P{y[+t7.7] w[+mC]=GMR48H10-GAL4}attP2<br>BL27390 y1 w*; P{w[+mC]=GAL4-Mef2.R}3<br>BL44277 w1118; P{y[+t7.7] w[+mC]=13XLexAop2-IVS-GCaMP6f-p10}su(Hw)attP5<br>BL5885 w*; P{w[+m*]=Sgs3-GFP}3<br>BL32219 w*; P{10XUAS-IVS-mCD8::RFP}attP40<br>BL55819 w1118; P{y[+t7.7] w[+mC]=8XLexAop2-FLPL}attP2<br>BL38879 P{w[+mC]=alphaTub84B(FRT.GAL80)}1, w*; Bl1/CyO; TM2/TM6B, Tb1<br>BL32199 w1118; P{10XUAS-IVS-myr::GFP}su(Hw)attP5<br>BL5138 y1 w*; P{w[+mC]=tubP-GAL4}LL7/TM3, Sb1 Ser1<br>BL7016 P{w[+mC]=tubP-GAL80[ts]}Sxl[9], w[*]/FM7c<br>BL80436 y1 v1; P{y[+t7.7] v[+t1.8]=TRiP.HMS06016}attP40<br>BL6871 w[1118]; P{w[+mC]=MsrA-GAL4.657}TP1-1 (Eip71CD-GAL4)<br>B28281 w*; P{w[+mC]=UAS-RedStinger}6, P{w[+mC]=UAS-FLP.Exel}3, P{w[+mC]=Ubi-p63E(FRT.STOP)Stinger}15F2 (G-TRACE stock110)
Wild animals	No wild animals were used in this study.
Field-collected samples	No field collected samples were used in this study.
Ethics oversight	No human data or cell lines, or vertebrate animals were used in this manuscript.

Note that full information on the approval of the study protocol must also be provided in the manuscript.
